# Children's Country Holidays Fund: Letter from the Bishop of London

**Published:** 1916-07-29

**Authors:** A. F. London

**Affiliations:** 18 Buckingham Street, Strand, W.C.


*To the Editor of* The Hospital.

### Children's Country Holidays Fund: Letter from the Bishop of London.

*To the Editor of* The Hospital.

Sir,—However opinion may be divided on the question of holidays in war-time, there is one section of the community to whom few of us would begrudge a happy fortnight in the country—the children of our big towns. Especially would I plead for the elementary school children of London, for many of whom the Children's Country Holidays Fund provides the only means of escape from their everyday surroundings during the hot summer months.

Workers amongst these children state that the war-heated atmosphere in which they live is telling its tale in overwrought nerves, strain, and restlessness; and local committees which began their work on a small scale now find their lists of ailing children much longer than they had anticipated.

As a result of the high cost of living, in many places the Fund has been obliged again to raise the weekly payment for board, and money is needed to meet the extra expense.

Cheques made payable to the Fund should be sent to 18 Buckingham Street, Strand.—Yours faithfully,

18 Buckingham Street, A. F. London.

Strand, W.C., July 19, 1916.

